# Corneal stromal mapping characteristics in normal corneas using anterior segment SD-OCT

**DOI:** 10.3389/fmed.2024.1485718

**Published:** 2024-12-02

**Authors:** Mohammad Abusamak, Sara Mazen Issa, Amal F. Alomari, Husam A. Alsalamat, Nour S. Haj Ali, Abdallah Izmegna, Mais Shawashreh, Mahmoud Abu Samak, Talal M. Abusamak

**Affiliations:** ^1^Department of Special Surgery, School of Medicine, Al-Balqa Applied University, Al-Salt, Jordan; ^2^Amman Eye Clinic, Amman, Jordan; ^3^Department of Biopharmaceutics and Clinical Pharmacy, School of Pharmacy, The University of Jordan, Amman, Jordan; ^4^Department of Clinical Pharmacy and Therapeutics, Applied Science Private University, Amman, Jordan; ^5^Kasralainy Faculty of Medicine, Cairo University, Cairo, Egypt

**Keywords:** stroma, ophthalmology, optical coherence tomography, mapping, anterior segment

## Abstract

This study investigated how normal corneal stromal profiles change with age, sex, and corrected intraocular pressure (IOP). A retrospective observational analytical study was conducted in Jordan, employing anterior segment spectral-domain optical coherence tomography AS-OCT, a measured corneal stromal thickness (CST) in 134 eyes across the central 6-mm corneal diameter. People between the ages of 18 and 79 were included, and the mean CST values were linked to age groups, IOP, and cis-gender populations, with the exclusion of certain eye conditions. The central stroma was thinnest at 484.6 (±32.6) μm, contrasting with the outer peripheral superior zone’s maximum thickness at 549.3 (±40.6) μm. A positive correlation between CST and the 30–49 age group was noted. In conclusion, this study highlights a centrifugal CST distribution, with the central stroma being the thinnest and the superior stroma being the thickest. AS SD-OCT, employing novel algorithms, proves vital in refractive surgery planning and corneal disease diagnosis. The research offers valuable insights into age, gender, and IOP interactions with corneal stromal characteristics, enhancing clinical strategies for corneal pathologies in the Jordanian population.

## 1 Introduction

In terms of structure, the cornea is composed of an avascular, collagen-rich stromal tissue lined by a self-renewing, stratified, non-keratinizing squamous epithelium that functions as a physical barrier between the eye’s internal structures and the outside world, thus protecting the eye from the external environment (1). The transparency of the cornea is dependent on the precise organization of the stroma proper. This orderly arrangement of uniform, small-diameter collagen fibrils is crucial for its transparency (2). In this regard, the absence of blood vessels, the unique architecture of collagen fibers, homogeneous extracellular matrix (ECM), and the relatively small number of stromal cells are all essential components (3).

Optical coherence tomography has been developed to reconstruct images of ocular structures including corneal thickness measurement, which has the advantages of non-contact, faster performance, and high reliability and repeatability (4–6). Moreover, the Anterior Segment OCT automatically generates corneal epithelial and stromal thickness maps, by which clinicians can obtain a wide-field evaluation of corneal thickness. OCT has become an essential clinical tool in ophthalmology. Cross-sectional images of the cornea, retina, choroid, and optic nerve can be obtained non-invasively by OCT. AS-OCT is used routinely for the diagnosis, evaluation of the progression of the disease, and monitoring the structural changes of the cornea before, during, and after treatments (4, 5, 7).

Studies have been done suggesting a link between central corneal thickness (CCT) and intraocular pressure (IOP), while some explained the findings as measurement errors due to overestimation of IOP in thicker corneas (8), others have concluded that CCT might be a powerful predictor of open angle glaucoma. Studies concerning corneal stromal thickness (CST) are a steppingstone to exploring this possible link and might help further ascertain central corneal thickness and CST as predictors of glaucoma.

Despite its clinical significance—add to the aforementioned, diagnosing keratoconus, pellucid degeneration, LASIK flap measurement, cross-linking depth assessment, trans-epithelial photorefractive keratectomy (Trans-PRK), corneal scar measurement in phototherapeutic keratectomy (PTK), and post-refractive surgery follow-up—the literature on the stromal thickness and its relationship to different variables such as age, sex, and IOP is scarce, and the studies that have been done deal with specific ethnicities and are limited by region. Given this, we considered it important to study stromal thickness. This, first of its type, study will increase our understanding of the normal stromal characteristics in Jordan and nearby Middle Eastern countries.

## 2 Materials and methods

### 2.1 Design and setting

This is a cross-sectional retrospective study that was conducted between January 2015 and December 2021 at the Amman Eye Clinic, a private ophthalmology center located in Amman, Jordan. A total of 134 eyes of 134 individuals out of 464 individuals examined were recruited for this study and provided consent to participate in the study, eye examination and consent to publication.

### 2.2 Sample size calculation

The G*Power (version 3.1.9.7) program was used to calculate the sample size, based on regression test. The minimum sample size required was 129 (power = 0.95, *α* = 0.05, medium effect size = 0.15, predictors = 4). A total of 134 were recruited for this study.

### 2.3 Eye selection procedure

Volunteers, patients seeking refractive surgery or cataract consultations, and patients seeking comprehensive ophthalmology examinations aged 18–79 were voluntarily enrolled in the study using a convenience sampling method (aka non-probability sampling). The study required the participants to meet the criteria for a normal cornea, defined by conducting a clinical examination and ruling out any exclusion criteria.

**Figure 1:**
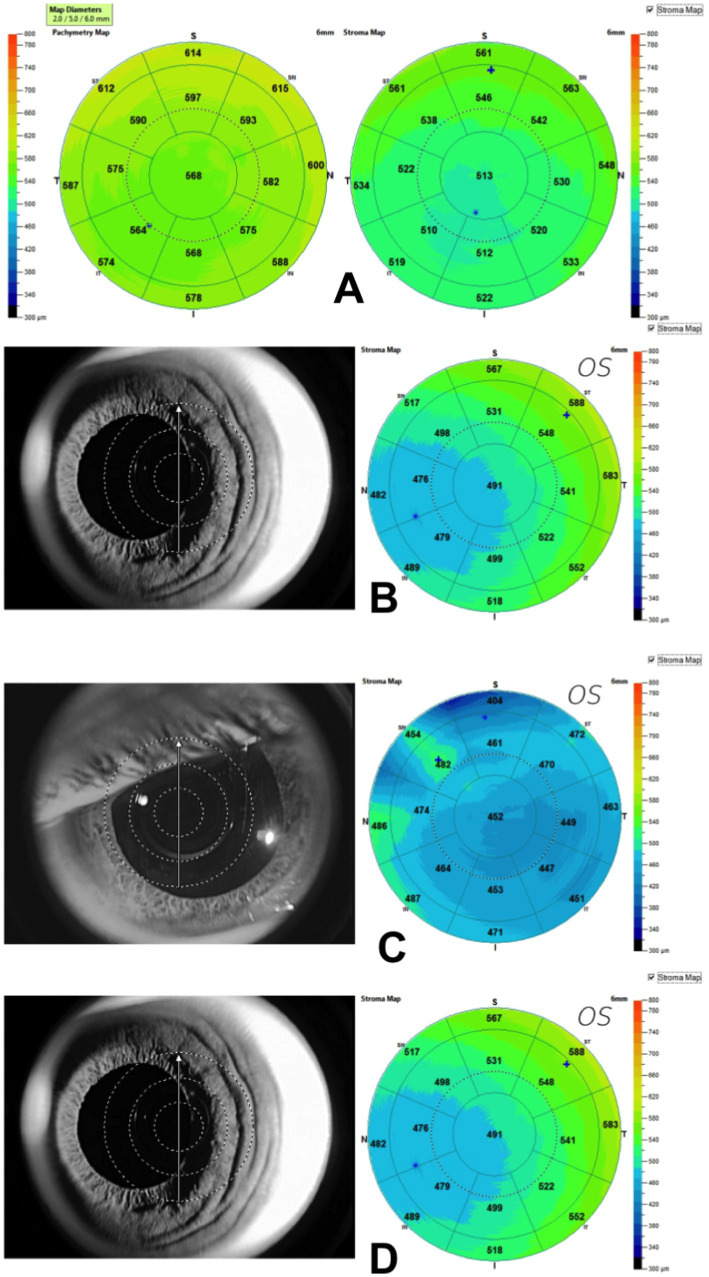
**(A)** Corneal Stromal Map (6 mm Ø) generated using Optovue Anterior segment OCT. Notice the non-homogeneous stromal surface as compared to the pachymetry surface. Examples of scans that were excluded. **(B)** A decentered scan of the left eye caused the corneal apex to shift to the nasal side; **(C)** Poor scan quality because of scan area coverage; **(D)** Subepithelial deposits caused by adenoviral keratitis in which the stroma is weakened; if the patient also has corneal ectasia, this condition could cause confusion.

We performed all ocular scans between 12:00 PM and 6:00 PM to account for daily fluctuations in pachymetric thickness (9–12). For statistical analysis, we selected only one eye based on the best signal strength index, lack of segmentation errors, image centration to pupil, and absence of the previously mentioned exclusion criteria. In case of both eyes met normal corneal parameters, and then a random selection was made (Figure 1A). We also performed a full ocular examination to rule out any corneal abnormalities, including corneal scarring, keratoconus, prior intraocular surgery, high myopia, and hyperopia, among others. The majority of cases (*n* = 133) used either subjective refraction or automated refraction (Topcon KR-8000, Tokyo, Japan).

### 2.4 Exclusion criteria

The medical records and scans of participants were examined for the following exclusion criteria: scans with inadequate corneal coverage, poor signal, scans that were off-centered (Figure 1B), or scans with a significant amount of irregularity (Figure 1C) (7). Previous intraocular surgery, contact lens wearing, post-refractive surgery, corneal inflammation (Figure 1D), dystrophy, dellen, pterygium, significant refractive errors (myopia >6D, hyperopia >4D, or astigmatism >3D), or glaucoma are all risk factors for these conditions. Scheimpflug tomography was used to identify keratectasia and keratoconus. To lessen the chance of causing harm to corneal epithelial cells, participants having tear film breakdown times less than 5 s, Schirmer I test results of less than 10 mm/5 min, or positive corneal staining/pooling were also disqualified. Macular scarring, age-related cataract, macular degeneration, newly diagnosed diabetic individuals, and retinitis pigmentosa were, nonetheless, included in the study (7). Additionally, all included scans were checked for signal strength index and corrected segmentation lines.

### 2.5 Keratoconus screening criteria

Finally, the keratoconus logistic regression model created by Qin et al. (9), was used to check each scan for keratoconus. As a result, each case with a high risk of developing keratoconus called for a repeated topography as well as, in some cases, a revision of the slit lamp and AS-OCT scans. We also disregarded cases of *forme fruste keratoconus* (FFK) or high risk for developing keratoconus.

### 2.6 Ethical approval

This study adhered to the principles of the Declaration of Helsinki and was approved by the Institutional Review Board at Applied Science University (2021-PHA-41). At the time of the initial clinic appointment, each subject’s and/or their guardian’s written informed consent was obtained for imaging, the measurement of corrected IOP, and the evaluation for dry eyes.

### 2.7 AS-OCT measurement of the CST

The CST measurements were taken using the add-on lens of the corneal adapter (CAM-L module, S/N 43412) and the anterior segment platform of the Optovue Avanti® 70,000 A-scans/second (Optovue, Fremont, CA, United States) spectral-domain OCT. There were eight meridional B-scans per capture, each with 1,024 A-scans and an axial resolution of 5 μm covering a 6 mm-diameter area. To avoid mistakes in corneal curvature or changes to the results, subjects were instructed to keep their eyes open wide while the scan was taking place. The operator made no attempt to open the subjects’ eyes. Within a few seconds of adequate fixation and centration, a scan was produced. The tear-film breakup time was reached before all readings were taken. Ophthalmic examinations and the Schirmer test were performed.

The corneal thickness maps were automatically created, with a total of 17 zones: one central zone with a diameter of 2 mm, eight paracentral zones making a circle 3 mm wide, and eight outer peripheral zones producing an outer circle 1 mm wide. The demographic and subject characteristic data were manually entered into Excel sheets where they were reviewed for accuracy and the existence of outliers or missing values.

### 2.8 Manifest and subject refraction

In total, 133 patients underwent automatic and subjective refraction. Axis, sphere, and cylinder were recorded.

### 2.9 IOP measurement

In the majority of patients (*n* = 101), Goldmann applanation tonometry was used, and it was corrected for the central corneal thickness (13, 14).

### 2.10 Statistical analysis

IBM SPSS Statistics for Mac, Ver. 28.0.1.0, was used to perform the statistical analysis (IBM Corporation, Armonk City, NY, United States). The continuous data were examined for normality and homogeneity using the Kolmogorov–Smirnov and Levene’s tests, respectively. The measured descriptive statistics included mean, range, and standard deviations. Inter-eye variability was examined by independent t-test in the central stromal zone and the ANOVA test was used to compare quantitative data between the various inner and outer zones to the center stromal thickness. A Holm-Bonferroni (HB) adjusted for three comparisons, for the center, inner, and outer zones in each octant, was used to reduce Type I error. To ascertain the impact of age, IOP, and sex on the stromal thickness in various zones of the stromal map, linear regression models were used. If there is a difference in stromal thickness between men and women, it was determined using a student’s independent t-test statistics. Keratometry (K), flat Keratometry (K), steep K, K average, sphere, cylinder, mean sphere equivalent, corneal curvatures, and stromal deviation values were additional variables included in the analysis.

## 3 Results

This section may be divided by subheadings. It should provide a concise and precise description of the experimental results, their interpretation, as well as the experimental conclusions that can be drawn.

### 3.1 Descriptive statistics and sample demographics

A total of 134 eyes of 134 individuals out of 464 examined individuals were selected upon meeting the criteria for normal cornea. The majority of final study participants were Jordanians (67.2%), Iraqis (23.1%), and other Arab Gulf region citizens (9.7%).

Detailed descriptive statistics performed on data obtained from the medical records and scan results are presented in Table 1. It includes refractive errors, IOP and demographic characteristics of the study population. A total of 134 individuals composed of 68 males (50.7%) and 66 females were included for analysis. The mean age was 44.7 ± 17.6 (male 44.3 ± 16.9, female 45.2 ± 18.3) ranging from 18 to 79 years, 41.8% of them were older than 50 years. In addition, these age groups had a lower magnitude of refractive errors that ranged between −0.51 D to 0.44 D and higher mean IOPs (16.4–18.2 mmHg). An independent *t*-test was performed to evaluate if there is a mean difference between right eye (*n* = 64) and left eye (*n* = 70) central stromal thickness; there was no statistically significant difference present (*p* = 0.796).

**Table 1:** Descriptive statistics of the study population per age group.

Age group in years	Patients, *n*	Sex, M/F	Refractive Error, SD	IOP, mmHg, SD
< 30	38	*17/21*	*−1.64 ± 1.8*	*15.5 ± 3.0*
			*n = 37*	*n = 20*
30–49	40	*25/15*	*−1.34 ± 1.8*	*16.7 ± 2.5*
			*n = 40*	*n = 36*
50–69	42	*22/20*	*−0.51 ± 1.9*	*16.4 ± 4.4*
			*n = 42*	*n = 33*
>70	14		*+0.44 ± 1.1*	*18.2 ± 2.7*
		*4/10*	*n = 14*	*n = 12*
Total	134	*66/68*	*−0.97 ± 1.9*	*16.6 ± 3.4*
			*N = 133*	*N = 101*

n, Count; IOP, Intra ocular pressure; SD, Standard deviation; mmHg, Millimeter mercury; M, Male; F, Female.

### 3.2 Stromal thickness in the 17 corneal zones

**Figure 2:**
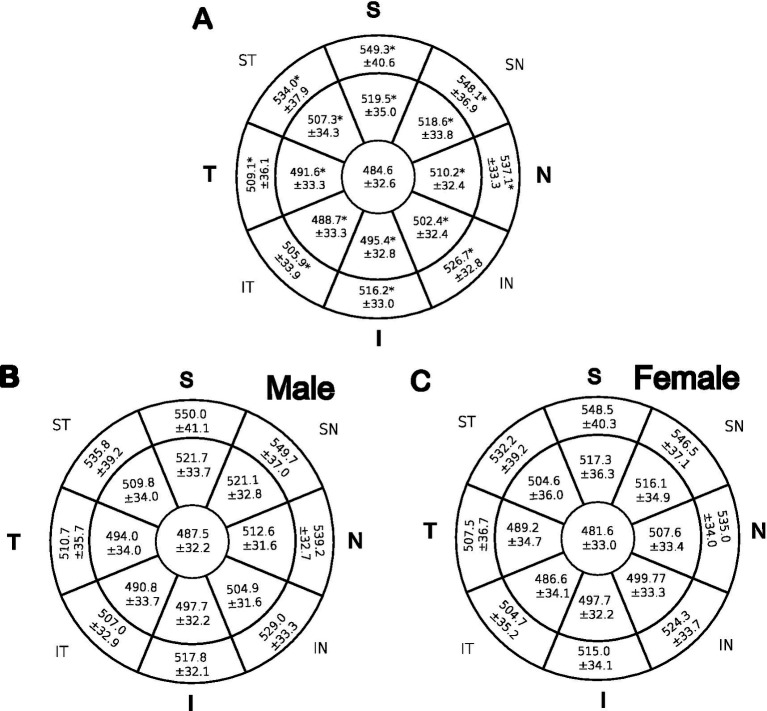
Mean CST in μm across 17 zones that make up eight octants. The means of each zone were compared using one-way ANOVA. **(A)**: represent all patients. Student *t*-test was performed for panel **(B)** males and **(C)** females. *indicates significant *p* values < 0.05.

The mean thicknesses of the center, inner, and outer zones of the stroma in eight octants are illustrated in Figure 2A. Superior and superior nasal quadrants showed the thickest stroma as compared to inferior and inferior temporal quadrants 13.4%, 13.1%, 6.6%, and 4.4%, respectively. The central stroma was the thinnest with a mean of 484.6 ± 32.6 μm, while the thickest point was the outer peripheral superior zone with a mean of 549.3 ± 40.6 μm. Variance analysis (one-way ANOVA) was used to examine whether the differences in the means of the stromal thickness maps were statistically significant in each of the 17 zones that comprise the 6 mm diameter circle. The mean thickness of the stroma increases from the center to the periphery in a centrifugal pattern, nasal zones were thicker than their temporal counterparts (*p* < 0.05). To test if the centrifugal increase in stromal thickness from center to periphery is statistically significant and not attributed to a type I error, both the size effect (eta-squared) and two *post-hoc* tests were performed: Bonferroni correction and a less strict sequential Holm-Bonferroni (HB) test with three comparisons per octant. As shown in Supplementary Table 1, the inferotemporal and temporal zones were likely caused by a type I error.

### 3.3 Gender

As illustrated, males (Figure 2B) showed thicker stroma than females (Figure 2C) in all zones examined by an independent *t*-test, for more statistical details refer to Supplementary Table 3. However, the difference was statistically insignificant (*p* > 0.05). Additionally, a linear regression analysis was conducted to determine whether there was a significant relationship between cis-gender within the same age group and between age groups of the same sex; no significant correlations were reported.

### 3.4 Effect of age on the stroma

A linear regression model was used to analyze the relationship between demographic factors such as age on stromal thickness, as shown in Supplementary Table 2. However, no statistically significant correlation between age as a continuous variable and stroma thickness was observed at *p* < 0.05. However, a negative, albeit insignificant, correlation between age and stromal thickness was observed in the central, inferior nasal, paracentral inferior, and paracentral inferior temporal zones, whereas all other zones demonstrated a positive, insignificant correlation.

Additionally, the age was divided into four sub-groups, and linear regression was used to analyze sub-group differences and correlations between age and stromal thickness. Interestingly, among those aged 30–49 years, 10 out of 17 stromal zones were statistically significant. In that particular cohort, the nasal quadrant resisted aging thinning alterations as demonstrated in Supplementary Table 2. The linear regression of the other age groups is presented in Supplementary Table 4.

Finally, the relationship between age and corneal stromal deviation was studied by the Pearson product–moment correlation coefficient, which led to the discovery that the determinant coefficient was significant (*r* = 0.235, *p* = 0.006). This indicates a moderately positive relationship between the two variables.

### 3.5 Intraocular pressure

Corrected IOP showed a strong correlation with stromal thickness in various stromal zones by linear regression as seen in Supplementary Table 2. Additionally, Supplementary Table 4 is a plot graph that demonstrates the negative linear regression correlation between the central stromal thickness and the corrected IOP.

## 4 Discussion

Corneal stromal thickness varies significantly between studies in normal adults, depending on factors like ethnic origin, sample demographics, refractive errors, diurnal variations, and age as seen in Table 2. Males had thicker stroma in all 17 zones, but the difference was not statistically significant. Furthermore, previous researchers, Figures 2B,C, supported this finding Table 2 (7, 12). We believe this observation is induced by sex hormones. This effect was demonstrated by the thickness of the corneal epithelia, which was significantly thicker in males compared to females (7, 13, 15). Gonadal hormone receptors are expressed in the nuclei of corneal epithelial, stromal, and endothelial cells (14, 16). Giuffra et al. (17) and Fortepiani et al. (18) demonstrated that the mean corneal thickness of women was thickest on the day of ovulation and thinnest at the end of the menstrual cycle. Interestingly, females showed more curved posterior cornea than males, this was reported in other research (19); one speculation is that it may be due to sex hormone levels and their fluctuation over time.

**Table 2:** Literature reported central stromal thickness in normal adults.

#	Author	Sample, *n* (M,F,All)	Age in years, Mean ± SD	ST in μm, Mean ± SD	AR in μm	Manufacture brand	Year
1	Kim et al. (15)	122 (M)	45.3 ± 18.0	489.6 ± 31.6	5	Optovue RTVue	2016
2	Kim et al. (15)	88 (F)	43.7 ± 17.3	484.4 ± 32.8	5	Optovue RTVue	2016
3	Optovue iVue FDA (31)	108 (All)	42.0 ± 15.7	485.2 ± 20.0	5	Optovue iVue	2017
4	Hashmani et al. (13)	118 (M)	40.0 (All)	467.4 ± 32.3	5	Optovue Avanti	2020
5	Hashmani et al. (13)	109 (F)		466.7 ± 30.1	5	Optovue Avanti	2020
6	Reinstein et al. (12)	110 eyes (56 All)	38.4 ± 12.0	465.4 ± 36.9	NA	Artemis VHFUS	2009
7	Haque et al. (32)	20 (9F, 11 M)	27.6 ± 5.9	463.4 ± 21.1	NA	Humphrey-Zeiss OCT 2000	2008
8	Batawi et al. (8)	58 (M)	68.2 ± 10.6	465.2 ± 31.2	4	Zeiss Cirrus OCT	2018
8	Luft et al. (33)	40 eyes (20 All)	33 6 ± 11	482.1 ± 25.0	4.16	Nidek	2016
9	Current study	68 (M)	44.3 ± 16.9	487.5 ± 32.2	5	Optovue Avanti	2022
10	Current study	66 (F)	45.2 ± 18.3	481.6 ± 33.0	5	Optovue Avanti	2022

*n, Sample number; ST, Stromal thickness; AR, Axial resolution; M, Male; F, Female; SD, Standard deviation; VHFUS, Very high frequency ultrasound; NA, Not available.*

Understanding the stromal thickness profile is fundamental for evaluating the biomechanics of the cornea in patients undergoing refractive surgery. Moreover, due to the fact that the following two conditions are linked with localized corneal thinning, normative stromal thickness profiles may prove to be a beneficial diagnostic tool for keratoconus or pellucid marginal degeneration (20, 21). It is essential that physicians investigate patients asking for surgery for risk factors that may alter the corneal thickness in certain patients, for instance in patients with sleep apnea, chronic hypoxia, and diabetic peripheral neuropathy (22–24).

Looking at the cornea at basic level may shed light on the normal and diseased eye. The normal human corneal stroma exhibits significant geometric anisotropy (25). Couple of previous studies applied *x*-ray scattering to plot the best-suited collagen orientation in normal corneas (26, 27). The preferred orthogonal orientation of the collagen was maintained in a vertical and horizontal pattern in the central cornea, where the collagen fibrils became circular or tangential in deposition at 1 mm from the limbus. The peripheral stromal region contains additional collagen lamellae and thus is thicker (25–27). This natural variation in corneal thickness has been linked to cell adhesion molecules like neurotrimin (NTM), contactin-associated protein-like 4 (CNTNAP4), and the collagen genes COL5A1 and COL8A2 (28).

Patel et al. (29) published a study in 2001 that studied the effect of aging on corneal tissues and discovered that keratocyte density decreased significantly as age increased at a rate of 0.45 percent per year. He did not, however, observe a significant correlation between aging and central stromal thickness. This may be explained in part by aging-related adaptations to the collagen fibrils, which thicken, as well as changes to the ECM (25, 26, 29) as well as keratocyte density decline as we age (13). Few studies have shown no correlation between age and stromal thickness, contrary to others where almost all zones had a significant negative correlation with age. At first, our study seems to be consistent with the former studies (10, 14, 20); however, in the current study, we observed that the stromal thickness of one particular age sub-group increased with age. As presented in Supplementary Table 2, individuals aged 30–49 years old exhibited a significant positive correlation with stromal thickness. We do not have an explanation for this observation, but it could be a result of hormonal changes and aging. Because the group sample size is 40 individuals, it may be prudent to refrain from drawing conclusions. Finally, the association between age and corneal stromal deviation was investigated, leading to the finding of a significant determinant coefficient. This implies that age and variations in stromal thickness have a relatively positive association (14).

Our study results, as explained in Supplementary Table 2, display a moderate correlation between ocular tonometry and CST. This is consistent with other published studies that indicate CST is more predictive of IOP than corneal epithelial thickness (10, 30).

## 5 Limitations

The small sample size and lack of random selection present some limitations in this study. Furthermore, the AS-OCT lacks the ability to differentiate between the endothelium cellular layer and the Descemet membrane, which might contribute to an overestimation of stromal thickness. Another possible limitation is that we did not study the physical characteristics of participants, including height, weight, and axial length.

## 6 Conclusion

In conclusion, using AS-OCT, this study establishes normative range of CST among patients in Jordan, and reveals a centrifugal CST distribution, where the thickest parts are found in the superior regions and the thinnest in the center region. Men have thicker stroma, which may be related to sex hormones. Reversals in corneal epithelial thickness and a favorable connection between stromal thickness and the 30–49-age range are two noteworthy findings.

## Data Availability

The original contributions presented in the study are included in the article/Supplementary material, further inquiries can be directed to the corresponding author.
