# Supplementary material for: Corneal stromal mapping characteristics in normal corneas using anterior segment SD-OCT
Source: Front Med (Lausanne). 2024 Dec 2;11:1485718. doi: 10.3389/fmed.2024.1485718 (PMC11646729; doi:10.3389/fmed.2024.1485718)
Supplement: Supplementary file 1 [file Data_Sheet_1.docx]

**Supplementary 1.** Stromal thicknesses mapping in all 17 zones, N = 134.

|  |  | ST in µm, Mean±SD | IQR | η² | *P* bonf | *P* holm |
| --- | --- | --- | --- | --- | --- | --- |
| Center |  | 484.6±32.6 | 41.5 |  | < .001*** | < .001*** |
| Superior | Inner | 519.5±35.0 | 46.8 | .35 | < .001*** | < .001*** |
|  |  |  |  |  |  |  |
|  | Outer | 549.3±40.6 | 55.0 |  | < .001*** | < .001*** |
|  |  |  |  |  |  |  |
| Superior-Nasal | Inner | 518.6±33.8 | 43.5 | .37 | < .001*** | < .001*** |
|  |  |  |  |  |  |  |
|  | Outer | 548.1±36.9 | 46.8 |  | < .001*** | < .001*** |
|  |  |  |  |  |  |  |
| Nasal | Inner | 510.2±32.5 | 43.8 | .30 | < .001*** | < .001*** |
|  |  |  |  |  |  |  |
|  | Outer | 537.1±33.3 | 45.8 |  | < .001*** | < .001*** |
|  |  |  |  |  |  |  |
| Inferior-Nasal | Inner | 502.4±32.4 | 45.0 | .22 | < .001*** | < .001*** |
|  |  |  |  |  |  |  |
|  | Outer | 526.7±32.8 | 43.8 |  | < .001*** | < .001*** |
|  |  |  |  |  |  |  |
| Inferior | Inner | 495.4±32.8 | 45.8 | .14 | < .02* | < .007** |
|  |  |  |  |  |  |  |
|  | Outer | 516.4±33.0 | 45.0 |  | < .001*** | < .001*** |
|  |  |  |  |  |  |  |
| Inferior-Temporal | Inner | 488.7±33.3 | 47.3 | .07 | 0.920 | 0.310 |
|  |  |  |  |  |  |  |
|  | Outer | 505.9±33.9 | 44.0 |  | < .001*** | < .001*** |
|  |  |  |  |  |  |  |
| Temporal | Inner | 491.6±34.3 | 48.5 | .08 | 0.280 | 0.090 |
|  |  |  |  |  |  |  |
|  | Outer | 509.1±36.1 | 46.8 |  | < .001*** | < .001*** |
|  |  |  |  |  |  |  |
| Superior-Temporal | Inner | 507.3±34.9 | 43.5 | .25 | < .001*** | < .001*** |
|  |  |  |  |  |  |  |
|  | Outer | 534.0±37.9 | 47.8 |  | < .001*** | < .001*** |
|  |  |  |  |  |  |  |

ST: Stromal thickness, SD: Standard deviation, IQR: Interquartile range. P-values are adjusted for comparing a family of 3 zones. η²: Eta-squared is an effect size measure. η²= 0.01 indicates a small effect. η² = 0.06 indicates a medium effect. η² = 0.14 indicates a large effect. * p < .05, ** p < .01, *** p < .001.

**Supplementary 2.** Linear regression of stromal thickness considering age and IOP.

|  | **All age groups in years (n = 134)** | | | **Age group 30 -49 years (n = 40)** | | | **IOP (n=134)** | | |
| --- | --- | --- | --- | --- | --- | --- | --- | --- | --- |
| **Stromal Zone** | **Regression Equation** | **R-value** | **P-value*** | **Regression Equation** | **R-value** | **P-value*** | **Regression Equation** | **R-value** | **P-value*** |
| **Central** | 484.8 - .004 x *age* | .002 | .979 | 418.95 +1.78 x *age* | .126 | .025* | 568.11 - 4.82 x *IOP* | .648 | < .001* |
| **Superior** |  |  |  |  |  |  |  |  |  |
| **Inner** | 511.6 + .171 x *age* | .084 | .333 | 454.61 + 1.734 x *age* | .112 | .035* | 606.42 - 5.00 x *IOP* | .616 | < .001* |
| **Outer** | 535.7 + .308 x *age* | .133 | .124 | 464.168 + 2.2 x *age* | .136 | .019* | 646.93 - 5.46 x *IOP* | .601 | < .001* |
| **Superior Nasal** |  |  |  |  |  |  |  |  |  |
| **Inner** | 515.2 + .076 x *age* | .039 | .652 | 458.08 + 1.643 x *age* | .108 | .039* | 603.67 - 4.84 x *IOP* | .626 | < .001* |
| **Outer** | 540.2 + .177 x *age* | .084 | .335 | 485.655 + 1.69 x *age* | .099 | .040* | 637.50 -5.05 x *IOP* | .601 | < .001* |
| **Nasal** |  |  |  |  |  |  |  |  |  |
| **Inner** | 511.6 - .032 x *age* | .017 | .842 | 457.71 + 1.454 x *age* | .087 | .064 | 593.33 - 4.76 x *IOP* | .639 | < .001* |
| **Outer** | 538.0 - .020 x *age* | .010 | .905 | 492.585 + 1.23 x *age* | .058 | .134 | 619.06 - 4.66 x *IOP* | .614 | < .001* |
| **Inferior Nasal** |  |  |  |  |  |  |  |  |  |
| **Inner** | 504.5 - .047 x *age* | .026 | .769 | 451.704 + 1.407 x *age* | .077 | .084 | 584.04 - 4.70 x *IOP* | .630 | < .001* |
| **Outer** | 527.1 - .008 x *age* | .004 | .960 | 486.609 +1.129 x *age* | .046* | .186 | 606.58 - 4.54 x *IOP* | .598 | < .001* |
| **Inferior** |  |  |  |  |  |  |  |  |  |
| **Inner** | 496.0 - .017 x *age* | .009 | .918 | 436.3 + 1.603 x *age* | .092 | .057 | 576.66 - 4.70 x *IOP* | .625 | < .001* |
| **Outer** | 516.0 + .010 x *age* | .005 | .952 | 459.588 + 1.52 x *age* | .079 | .080 | 599.15 - 4.75 x *IOP* | .626 | < .001* |
| **Inferior temporal** |  |  |  |  |  |  |  |  |  |
| **Inner** | 489.5 - .016 x *age* | .009 | .921 | 422.472 + 1.791 x *age* | .111 | .036* | 572.31 - 4.86 x *IOP* | .636 | < .001* |
| **Outer** | 505.0 + .108 x *age* | .009 | .915 | 436.306 + 1.843 x *age* | .111 | .036* | 588.81 - 4.82 x *IOP* | .617 | < .001* |
| **Temporal** |  |  |  |  |  |  |  |  |  |
| **Inner** | 489.9 + .039 x *age* | .020 | .818 | 489.9 + .039 x *age* | .121 | .028* | 577.28 - 5.00 x *IOP* | .634 | < .001* |
| **Outer** | 505.0 + .091 x *age* | .044 | .610 | 435.784 + 1.932 x *age* | .114 | .033* | 595.94 - 5.07 x *IOP* | .610 | < .001* |
| **Superior Temporal** |  |  |  |  |  |  |  |  |  |
| **Inner** | 501.8 + .123 x *age* | .062 | .478 | 437.307 + 1.846 x *age* | .122 | .027* | 594.24 - 5.03 x *IOP* | .625 | < .001* |
| **Outer** | 525.0 + .203 x *age* | .094 | .281 | 463.306 + 1.804 x *age* | .105 | .042* | 624.58 - 5.23 x *IOP* | .595 | < .001* |

IOP: Intraocular pressure. * Indicates *p-value* < .05; R indicates the correlation coefficient.

**Supplementary 3.**

| Table Idependet t-test Gender Stromal thickness | | | | | | |
| --- | --- | --- | --- | --- | --- | --- |
| **Zone/Gender** |  | **Sex** | | | |  |
|  |  | **Female *n* = 66** | | **Male  *n* = 68** | | ****p*** < .05 |
|  |  | **Mean (µm)** | **SD** | **Mean (µm)** | **SD** |  |
| **Center** |  | 481.7 | 32.9 | 487.5 | 32.2 | 0.758 |
| **Superior** | Inner | 516.5 | 35.7 | 521.9 | 33.9 | 0.55 |
|  | Outer | 546.8 | 40.1 | 550.2 | 41.5 | 0.757 |
| **Superior-Nasal** | Inner | 508.2 | 37.3 | 514.6 | 33.3 | 0.263 |
|  | Outer | 535.9 | 39.8 | 542.4 | 35.2 | 0.175 |
| **Nasal** | Inner | 495.5 | 37.2 | 500.8 | 33.5 | 0.392 |
|  | Outer | 516.5 | 40.3 | 520.5 | 35.8 | 0.267 |
| **Inferior-Nasal** | Inner | 490.9 | 35.8 | 495.7 | 32.3 | 0.342 |
|  | Outer | 510 | 37.7 | 513.7 | 33.2 | 0.267 |
| **Inferior** | Inner | 492.9 | 33.2 | 497.8 | 32.4 | 0.683 |
|  | Outer | 514.4 | 33.5 | 518.1 | 32.6 | 0.789 |
| **Inferior-Temporal** | Inner | 495.4 | 32.4 | 500 | 33.4 | 0.909 |
|  | Outer | 518.2 | 33.2 | 522.3 | 34.9 | 0.63 |
| **Temporal** | Inner | 501.2 | 32.8 | 505.5 | 34.2 | 0.746 |
|  | Outer | 525.7 | 34.9 | 528.8 | 37 | 0.438 |
| **Superior-Temporal** | Inner | 511.6 | 33.5 | 516.4 | 34.3 | 0.788 |
|  | Outer | 541.2 | 36.3 | 543.1 | 39.6 | 0.558 |

**Supplementary 4.**

| Table (4A) Linear regression of stromal zones with age group < 30 years (***n*** = 38) | | | |
| --- | --- | --- | --- |
| **Stromal Zone** | **Regression Equation** | **R^2^-value** | **P-value*** |
| **Central** | 437.1+ 2.0 x *age* | 0.043 | 0.214 |
| **Superior** |  |  |  |
| **Inner** | 456.568 + 2.578 x *age* | 0.063 | 0.128 |
| **Outer** | 508.47 + 1.5 x *age* | 0.016 | 0.455 |
| **Superior Nasal** |  |  |  |
| **Inner** | 453.00 + 2.77 x *age* | 0.073 | 0.101 |
| **Outer** | 476.147 + 2.943 x *age* | 0.068 | 0.114 |
| **Nasal** |  |  |  |
| **Inner** | 450.76 + 2.581 x *age* | 0.067 | 0.116 |
| **Outer** | 471.868 + 2.849 x *age* | 0.076 | 0.093 |
| **Inferior Nasal** |  |  |  |
| **Inner** | 453.311 + 2.149 x *age* | 0.018 | 0.203 |
| **Outer** | 484.266 + 1.833 x *age* | 0.032 | 0.282 |
| **Inferior** |  |  |  |
| **Inner** | 456.67 + 1.67 x *age* | 0.026 | 0.334 |
| **Outer** | 487.344 + 1.29 x *age* | 0.014 | 0.481 |
| **Inferior Temporal** |  |  |  |
| **Inner** | 447.39 + 1.78 x *age* | 0.03 | 0.297 |
| **Outer** | 467.246 + 1.655 x *age* | 0.024 | 0.35 |
| **Temporal** |  |  |  |
| **Inner** | 443.554 + 1.948 x *age* | 0.037 | 0.455 |
| **Outer** | 464.486 + 1.863 x *age* | 0.032 | 0.284 |
| **Superior Temporal** |  |  |  |
| **Inner** | 450.4 +2.364 x *age* | 0.0555 | 0.158 |
| **Outer** | 479.578 + 2.203 x *age* | 0.042 | 0.217 |
| * Indicates *p*  < .05 | | | |

**Supplementary 5.**

| Table (4C) Linear regression of stromal zones with age group 50 - 69 years (***n*** = 42) | | | |
| --- | --- | --- | --- |
| **Stromal Zone** | **Regression Equation** | **R^2^-value** | **P-value*** |
| **Central** | 460.0 + .305 x *age* | 0.003 | 0.751 |
| **Superior** |  |  |  |
| **Inner** | 466.87 +.812 x *age* | 0.015 | 0.439 |
| **Outer** | 511.34 + .577 x *age* | 0.007 | 0.611 |
| **Superior Nasal** |  |  |  |
| **Inner** | 473.64 + .662 x *age* | 0.022 | 0.513 |
| **Outer** | 491.194 + .883 x *age* | 0.016 | 0.421 |
| **Nasal** |  |  |  |
| **Inner** | 477.86 + .433 x *age* | 0.005 | 0.649 |
| **Outer** | 503.524 + .459 x *age* | 0.008 | 0.574 |
| **Inferior Nasal** |  |  |  |
| **Inner** | 469.73 + .438 x *age* | 0.006 | 0.635 |
| **Outer** | 490.273 + .518 x *age* | 0.008 | 0.574 |
| **Inferior** |  |  |  |
| **Inner** | 465.23 + .408 x *age* | 0.005 | 0.657 |
| **Outer** | 488.311 + .383 x *age* | 0.004 | 0.673 |
| **Inferior Temporal** |  |  |  |
| **Inner** | 462.582 + .337 x *age* | 0.003 | 0.721 |
| **Outer** | 484.452 + .266 x *age* | 0.002 | 0.78 |
| **Temporal** |  |  |  |
| **Inner** | 461.139 + .414 x *age* | 0.004 | 0.678 |
| **Outer** | 468.775 + .596 x *age* | 0.008 | 0.564 |
| **Superior Temporal** |  |  |  |
| **Inner** | 463.992 + .655 x *age* | 0.01 | 0.531 |
| **Outer** | 481.043 + .838 x *age* | 0.014 | 0.457 |
| * Indicates *p*  < .05 | | | |

**Supplementary 6.**

| Table (4D) Linear regression of stromal zones with age group 70+ years (***n*** = 14 ) | | | |
| --- | --- | --- | --- |
| **Stromal Zone** | **Regression Equation** | **R^2^-value** | **P-value*** |
| **Central** | 268.8 + 2.94 x *age* | 0.114 | 0.239 |
| **Superior** |  |  |  |
| **Inner** | 325.12 + 3.012 x *age* | 0.065 | 0.38 |
| **Outer** | 489.133 + 1.026 x *age* | 0.004 | 0.82 |
| **Superior Nasal** |  |  |  |
| **Inner** | 304.0 + 2.97 x *age* | 0.1 | 0.271 |
| **Outer** | 384.135 +2.329 x *age* | 0.047 | 0.457 |
| **Nasal** |  |  |  |
| **Inner** | 321.51 - 2.56 x *age* | 0.087 | 0.307 |
| **Outer** | 368.341 +2.313 x *age* | 0.063 | 0.386 |
| **Inferior Nasal** |  |  |  |
| **Inner** | 348.37 + 2.09 x *age* | 0.068 | 0.369 |
| **Outer** | 416.893 + 1.518 x *age* | 0.032 | 0.541 |
| **Inferior** |  |  |  |
| **Inner** | 365.69 + 1.76 x *age* | 0.055 | 0.419 |
| **Outer** | 446.462 + .986 x *age* | 0.016 | 0.664 |
| **Inferior Temporal** |  |  |  |
| **Inner** | 489.5 - .016 x *age* | 0.092 | 0.291 |
| **Outer** | 295.847 + 4.331 x *age* | 0.11 | 0.243 |
| **Temporal** |  |  |  |
| **Inner** | 221.197 +3.701 x *age* | 0.123 | 0.22 |
| **Outer** | 194.077 + 4.3311 x *age* | 0.125 | 0.215 |
| **Superior Temporal** |  |  |  |
| **Inner** | 261.104 + 3.411 x *age* | 0.09 | 0.295 |
| **Outer** | 284.35 + 2.821 x *age* | 0.043 | 0.478 |
| * Indicates *p*  < .05 | | | |

**Supplementary 7.**

| Table (5) Linear regression of stromal zones with age group 30 -49 years (***n*** = 40) | | | |
| --- | --- | --- | --- |
| **Stromal Zone** | **Regression Equation** | **R^2^-value** | **P-value*** |
| **Central** | 418.95 +1.78 x *age* | 0.126 | 0.025* |
| **Superior** |  |  |  |
| **Inner** | 454.61 + 1.734 x *age* | 0.112 | 0.035* |
| **Outer** | 464.168 + 2.2 x *age* | 0.136 | .019* |
| **Superior Nasal** |  |  |  |
| **Inner** | 458.08 + 1.643 x *age* | 0.108 | .039* |
| **Outer** | 485.655 + 1.69 x *age* | 0.099 | 0.04 |
| **Nasal** |  |  |  |
| **Inner** | 457.71 + 1.454 x *age* | 0.087 | 0.064 |
| **Outer** | 492.585 + 1.23 x *age* | 0.058 | 0.134 |
| **Inferior Nasal** |  |  |  |
| **Inner** | 451.704 + 1.407 x *age* | 0.077 | 0.084 |
| **Outer** | 486.609 +1.129 x *age* | 0.046 | 0.186 |
| **Inferior** |  |  |  |
| **Inner** | 436.3 + 1.603 x *age* | 0.092 | 0.057 |
| **Outer** | 459.588 + 1.52 x *age* | 0.079 | 0.08 |
| **Inferior Temporal** |  |  |  |
| **Inner** | 422.472 + 1.791 x *age* | 0.111 | 0.036* |
| **Outer** | 436.306 + 1.843 x *age* | 0.111 | .036* |
| **Temporal** |  |  |  |
| **Inner** | 489.9 + .039 x *age* | 0.121 | 0.028* |
| **Outer** | 435.784 + 1.932 x *age* | 0.114 | .033* |
| **Superior Temporal** |  |  |  |
| **Inner** | 437.307 + 1.846 x *age* | 0.1222 | 0.027* |
| **Outer** | 463.306 + 1.804 x *age* | 0.105 | .042* |
| * Indicates *p*  < .05 | | | |

**Supplementary 8.**


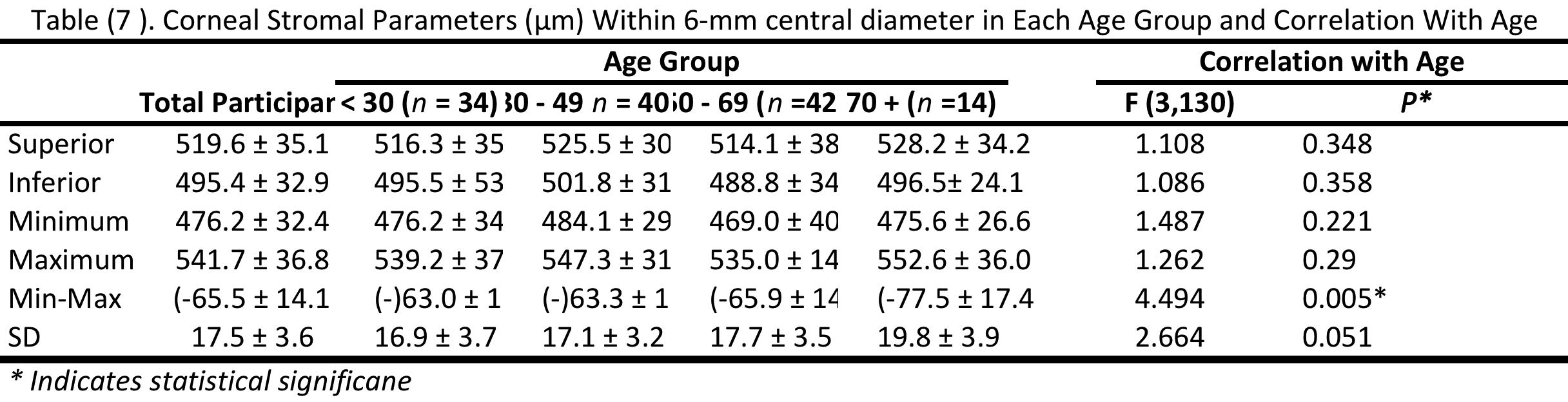


| Table ( ). Corneal Stromal Parameters (μm) Within 6-mm central diameter in Each Age Group and Correlation With Age | | | | | | | | |
| --- | --- | --- | --- | --- | --- | --- | --- | --- |
|  |  | **Age Group** | | | |  | **Correlation with Age** | |
|  | **Total Participants** | **< 30 (***n* **= 34)** | **30 - 49** *n* **= 40)** | **50 - 69 (***n* **=42)** | **70 + (***n* **=14)** |  | **F (3,130)** | ***P**** |
| Superior | 519.6 ± 35.1 | 516.3 ± 35.7 | 525.5 ± 30.4 | 514.1 ± 38.6 | 528.2 ± 34.2 |  | 1.108 | 0.348 |
| Inferior | 495.4 ± 32.9 | 495.5 ± 536.1 | 501.8 ± 31.0 | 488.8 ± 34.0 | 496.5± 24.1 |  | 1.086 | 0.358 |
| Minimum | 476.2 ± 32.4 | 476.2 ± 34.0 | 484.1 ± 29.4 | 469.0 ± 40.6 | 475.6 ± 26.6 |  | 1.487 | 0.221 |
| Maximum | 541.7 ± 36.8 | 539.2 ± 37.8 | 547.3 ± 31.1 | 535.0 ± 14.0 | 552.6 ± 36.0 |  | 1.262 | 0.29 |
| Min-Max | (-65.5 ± 14.1 | (-)63.0 ± 13.3 | (-)63.3 ± 11.6 | (-65.9 ± 14.0 | (-77.5 ± 17.4 |  | 4.494 | 0.005* |
| SD | 17.5 ± 3.6 | 16.9 ± 3.7 | 17.1 ± 3.2 | 17.7 ± 3.5 | 19.8 ± 3.9 |  | 2.664 | 0.051 |
| ** Indicates statistical significane* | | | | | | | | |


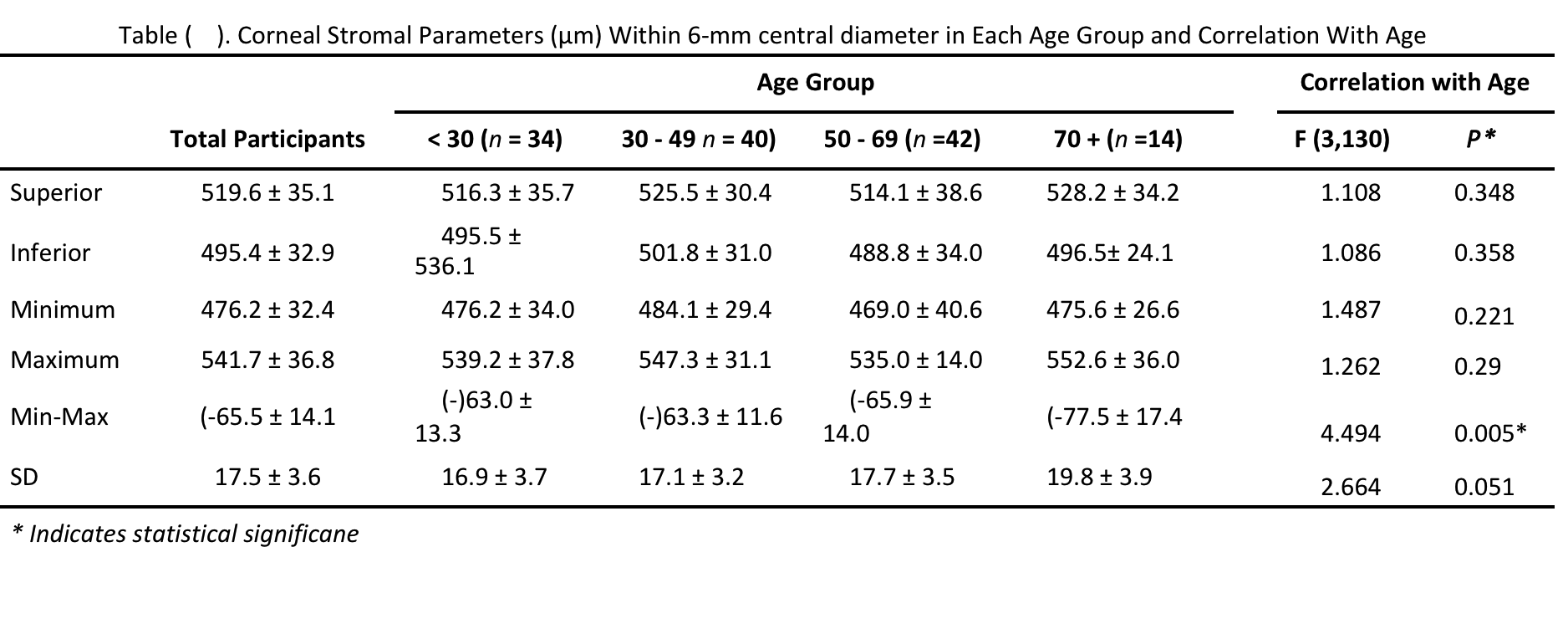


**Supplementary 9**

| Independent Samples T-Test Corneal True Power and curvature by Sex, N = 134 | | | | | |
| --- | --- | --- | --- | --- | --- |
|  |  | Male n = 68 |  | Female  n = 66 | p < .05 |
| Corneal Parameter | | Mean ± SD |  | Mean ± SD |  |
| Net Corneal True Power | | 42.5 ± 1.5 D |  | 43.0 ± 1.5 D | 0.053 |
| Corneal Power Anterior | | 48.4 ± 1.7 D |  | 49.0 ± 1.7 D | 0.053 |
| Corneal Power Posterior | | -6.0 ± 0.2 D |  | -6.1 ± 0.2 D | 0.016* |
| Anterior Curvature Radius | | 7.8 ± 0.3 mm |  | 7.7 ± 0.3 mm | 0.053 |
| Posterior Curvature Radius | | 6.6 ± 0.3 mm |  | 6.5 ± 0.2 mm | 0.016* |
| Note.  Student's t-test. * p < .05 | | | | | |

**Supplementary 10**

1. Regression plots of Corrected IOP and Corneal stroma thickness at 17 zones.


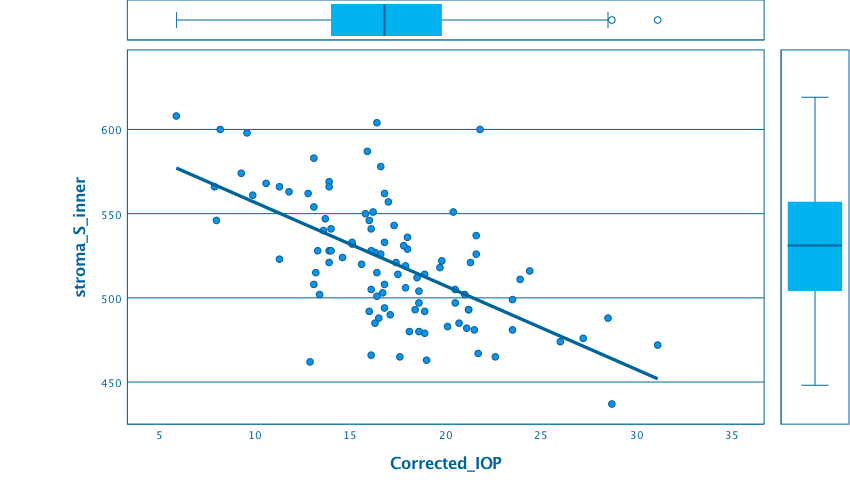


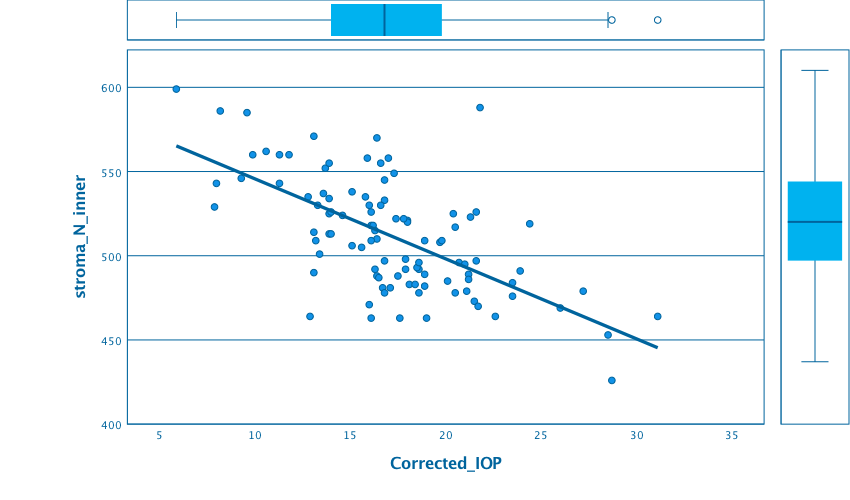


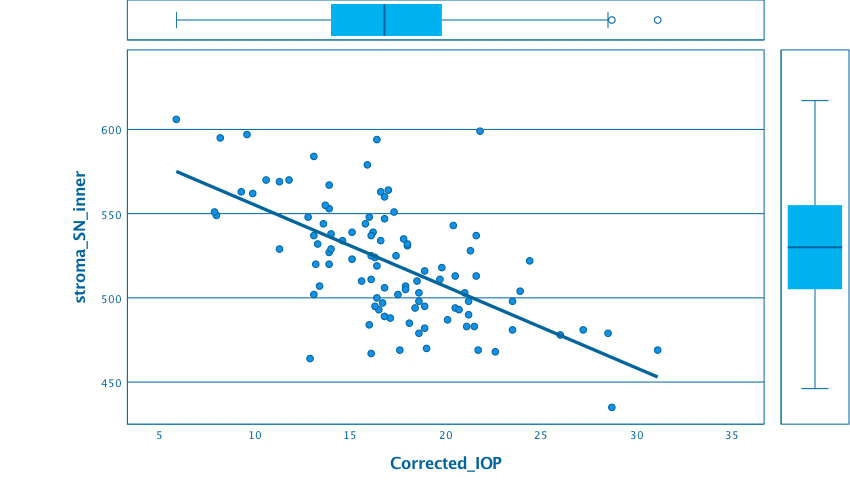


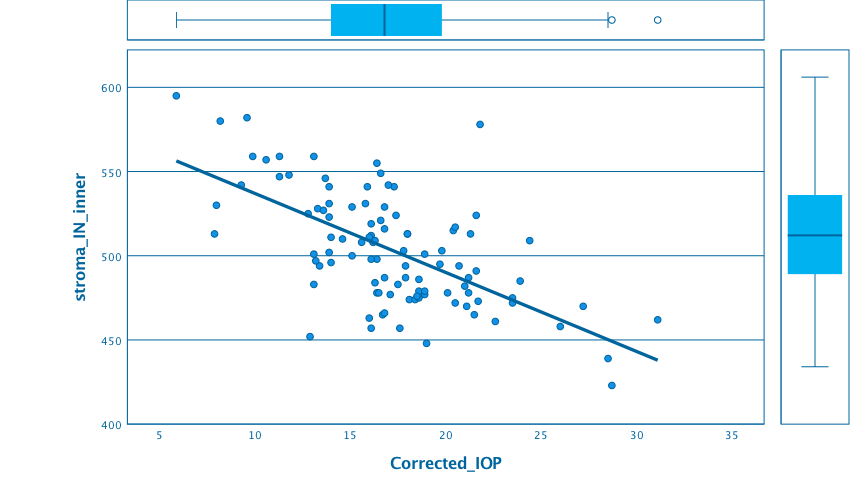


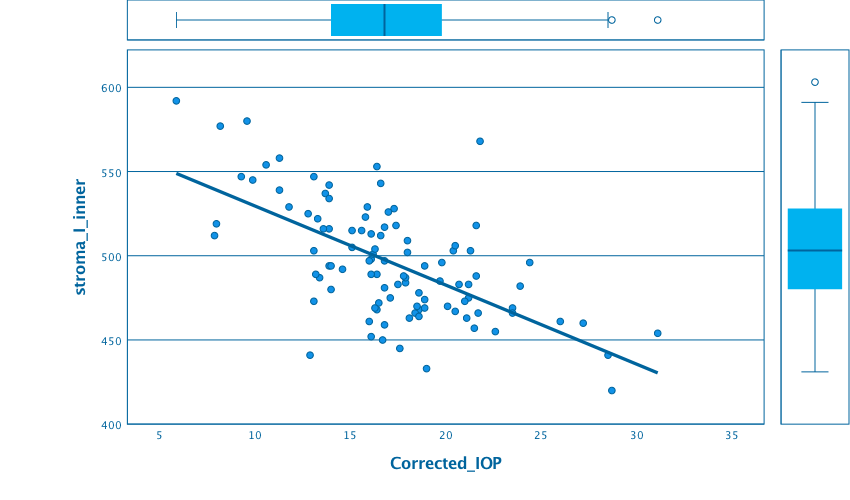


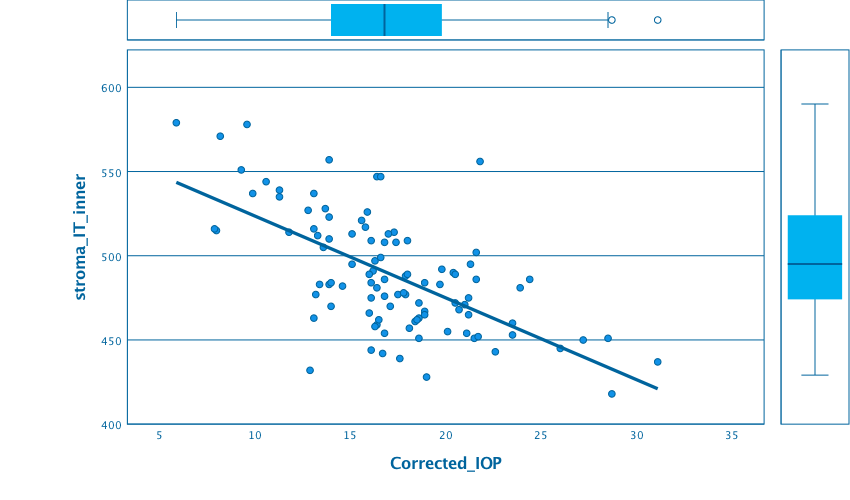


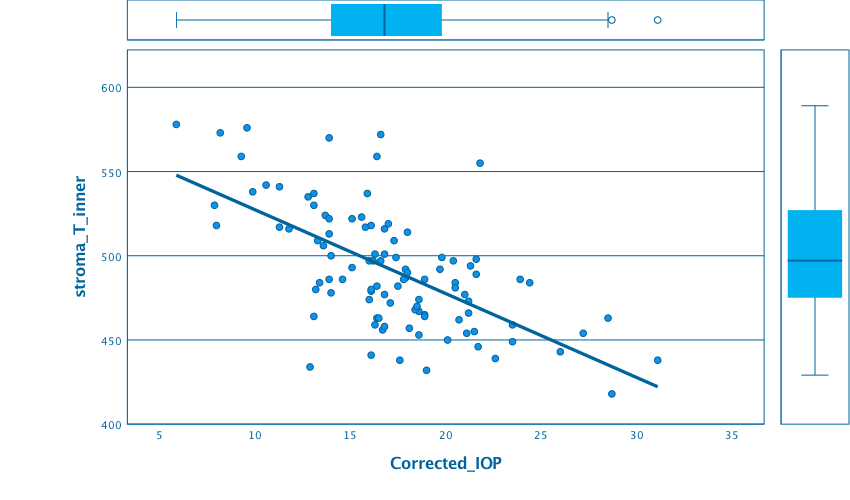


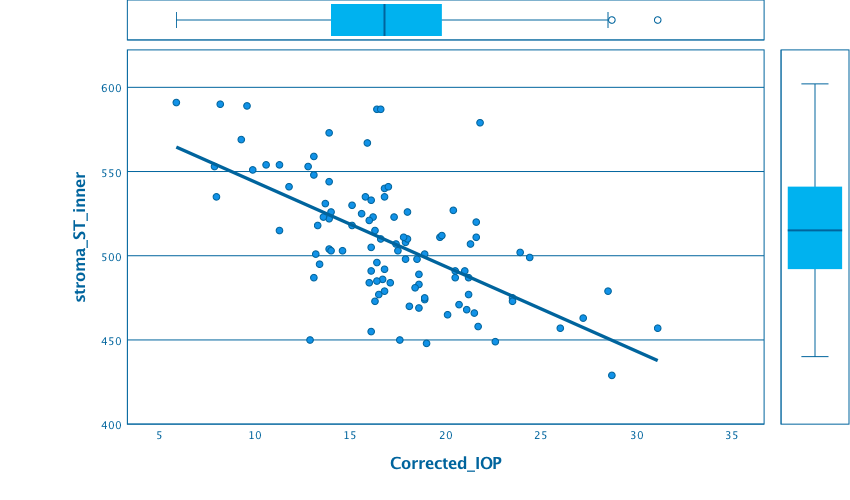


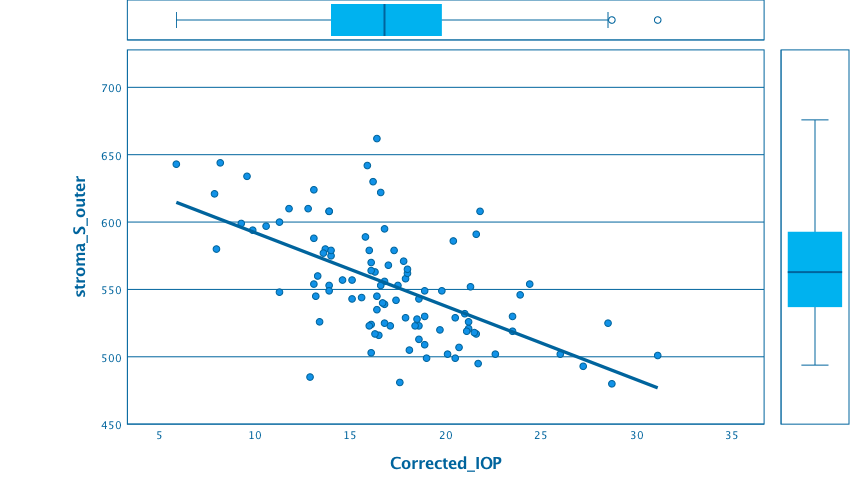


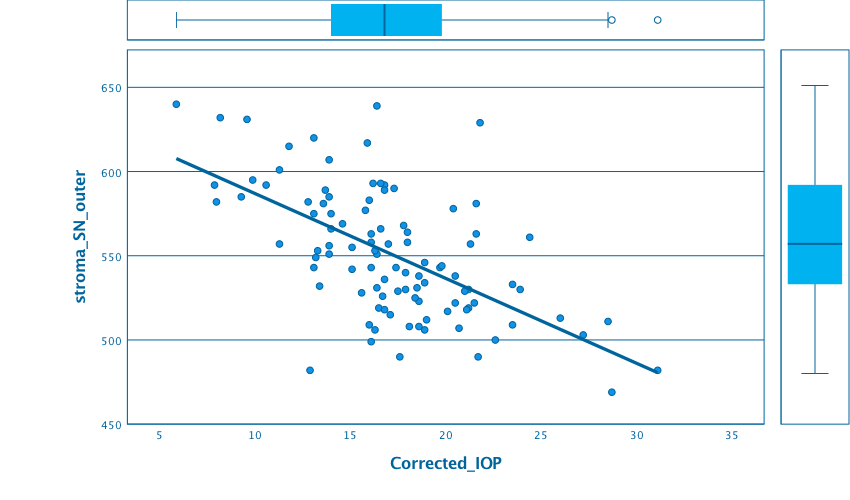


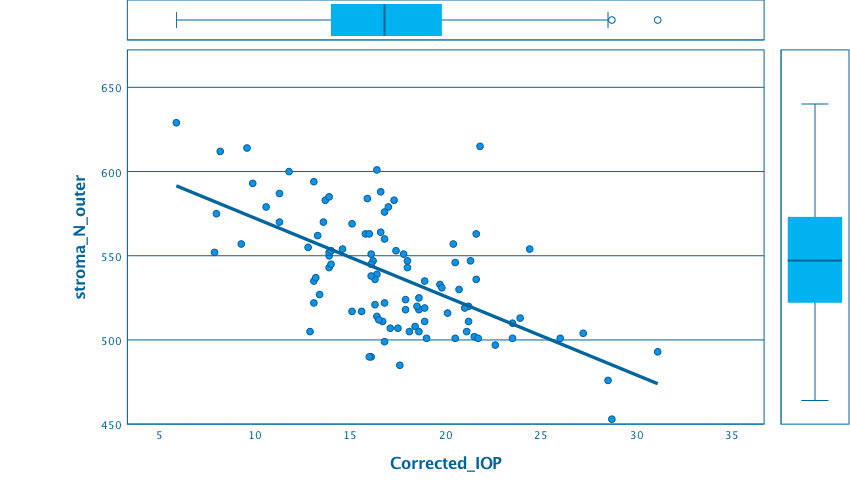


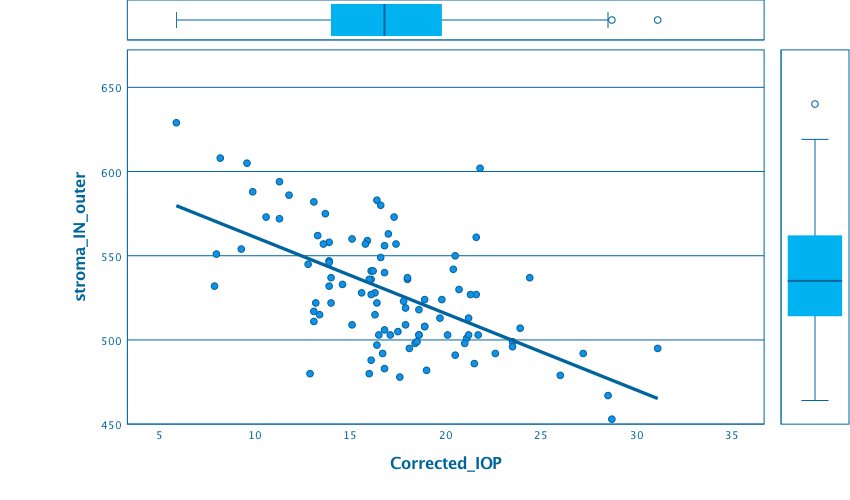


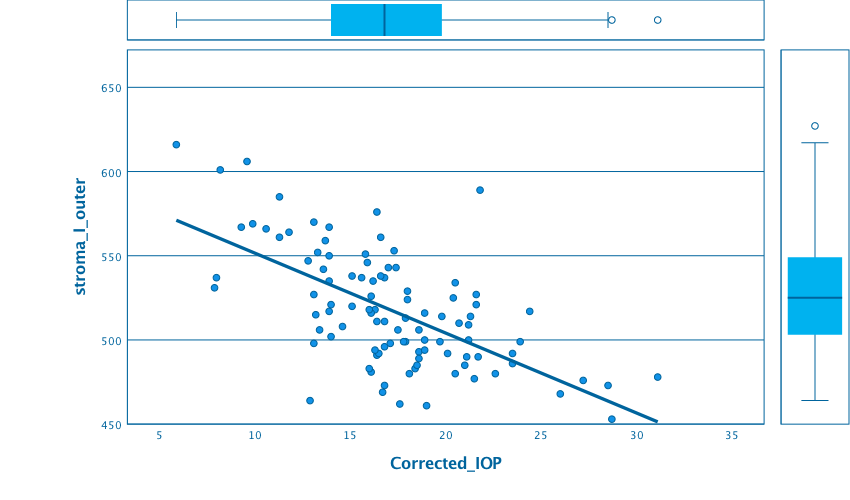


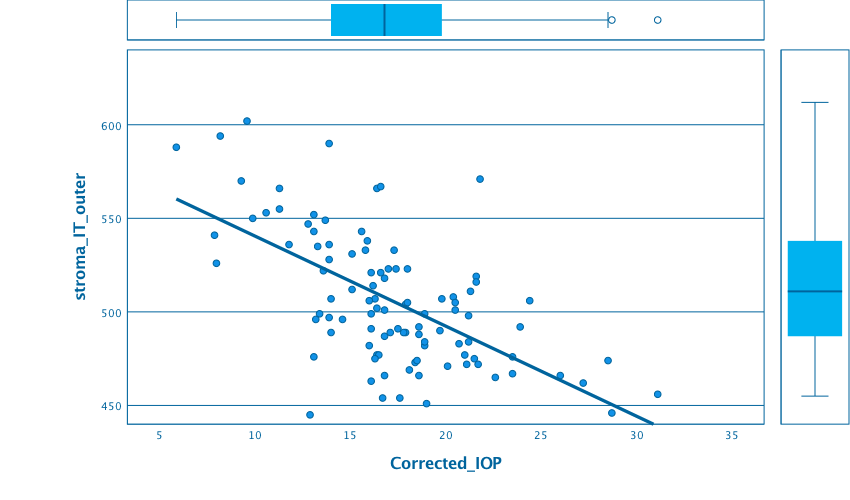

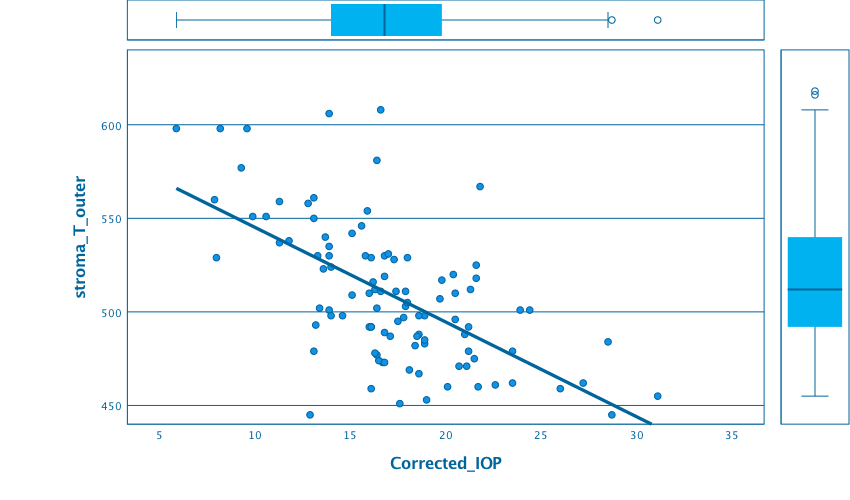


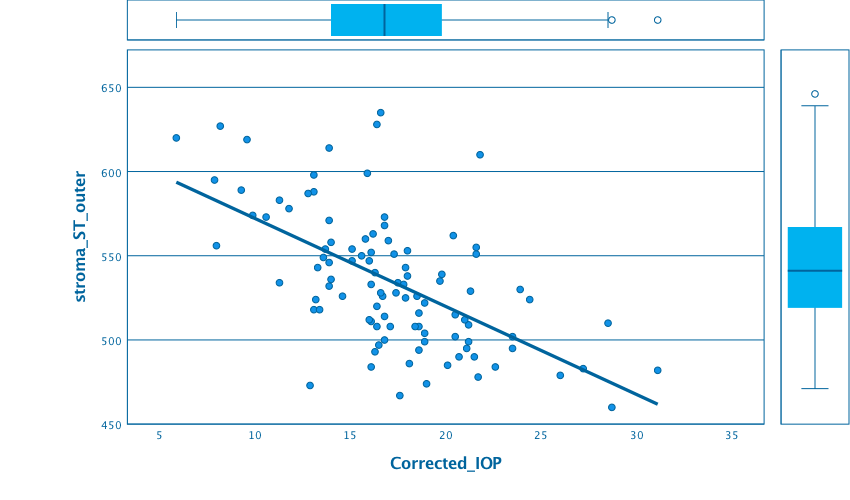


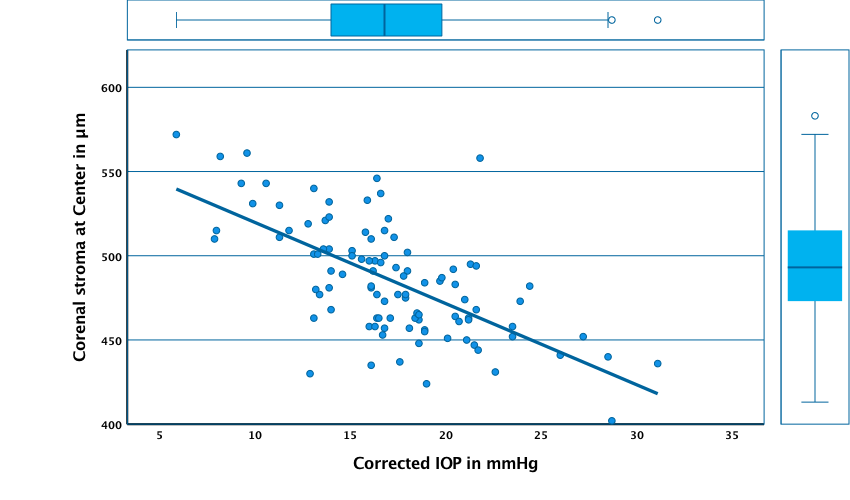


Linear regression plot graph between central corneal stroma and corrected IOP.

**Supplementary 11: Descriptive statistics of Corneal variables**

| Variable | Mean | Std. Dev. | Min | Max |  |
| --- | --- | --- | --- | --- | --- |
| CP Net | 42.775 | 1.537 | 39.14 | 47.58 |  |
| CP Anterior | 48.728 | 1.717 | 44.68 | 54.3 |  |
| CP Posterior | -6.081 | .233 | -6.86 | -5.54 |  |
| Curvature Ant Radius | 7.726 | .273 | 6.924 | 8.416 |  |
| Curvature Post Radius | 6.587 | .252 | 5.828 | 7.224 |  |
| Pachy offset SN-IT | 30.53 | 13.312 | -10 | 68 |  |
| Pachy offset SI | 21.903 | 14.224 | -36 | 55 |  |
| Pachy offset Min | 530.418 | 33.073 | 451 | 615 |  |
| Pachy offset Location Y | -444.663 | 389.394 | -2365 | 618 |  |
| Pachy offset Median | -22.642 | 5.975 | -34 | 21 |  |
| Pachy offset Min Max | -64.201 | 13.599 | -103 | -35 |  |
| CP: Corneal Power. Ant. : Anterior. Post.: Posterior. Pachy.: Pachymetry. Min.: Minimum. Max.: Maximum. | | | | | |
